# High HIV incidence in the postpartum period sustains vertical transmission in settings with generalized epidemics: a cohort study in Southern Mozambique

**DOI:** 10.7448/IAS.17.1.18808

**Published:** 2014-03-05

**Authors:** Caroline De Schacht, Nédio Mabunda, Orlando C Ferreira, Nália Ismael, Nurbai Calú, Iolanda Santos, Heather J Hoffman, Catharina Alons, Laura Guay, Ilesh V Jani

**Affiliations:** 1 Elizabeth Glaser Pediatric AIDS Foundation, Maputo, Mozambique; 2 Instituto Nacional de Saúde, Ministry of Health, Maputo, Mozambique; 3 Biology Institute, Federal University of Rio de Janeiro, Rio de Janeiro, Brazil;; 4 Provincial Health Directorate Gaza, Xai-Xai, Mozambique; 5 Provincial Health Directorate Maputo, Matola, Mozambique; 6 Department of Epidemiology and Biostatistics, School of Public Health and Health Services, The George Washington University, Washington DC, USA

**Keywords:** PMTCT, breastfeeding, incidence, HIV, elimination paediatric HIV, Mozambique

## Abstract

**Introduction:**

Acute infection with HIV in the postpartum period results in a high risk of vertical transmission through breastfeeding. A study was done to determine the HIV incidence rate and associated risk factors among postpartum women in Southern Mozambique, where HIV prevalence among pregnant women is 21%.

**Methods:**

A prospective cohort study was conducted in six rural health facilities in Gaza and Maputo provinces from March 2008 to July 2011. A total of 1221 women who were HIV-negative on testing at delivery or within two months postpartum were recruited and followed until 18 months postpartum. HIV testing, collection of dried blood spot samples and administration of a structured questionnaire to women were performed every three months. Infant testing by DNA-PCR was done as soon as possible after identification of a new infection in women. HIV incidence was estimated, and potential risk factors at baseline were compared using Poisson regression.

**Results:**

Data from 957 women were analyzed with follow-up after the enrolment visit, with a median follow-up of 18.2 months. The HIV incidence in postpartum women is estimated at 3.20/100 women-years (95% CI: 2.30–4.46), with the highest rate among 18- to 19-year-olds (4.92 per 100 women-years; 95% CI: 2.65–9.15). Of the new infections, 14 (34%) were identified during the first six months postpartum, 11 (27%) between 6 and 12 months and 16 (39%) between 12 and 18 months postpartum. Risk factors for incident HIV infection include young age, low number of children, higher education level of the woman's partner and having had sex with someone other than one's partner. The vertical transmission was 21% (95% CI: 5–36) among newly infected women.

**Conclusions:**

Incidence of HIV is high among breastfeeding women in Southern Mozambique, contributing to increasing numbers of HIV-infected infants. Comprehensive primary prevention strategies targeting women of reproductive age, particularly pregnant and postpartum women and their partners, will be crucial for the elimination of paediatric AIDS in Africa.

## Introduction

In 2012, 2.3 million new HIV infections occurred worldwide with 1.6 million of these in sub-Saharan Africa. Every day, about 6300 adults are infected globally, of which 34% are women aged 15–24 years [1].

Maternal acquisition of HIV during the breastfeeding period and subsequent risk of transmission to the infant places a high burden on efforts to eliminate paediatric HIV infection [2]. Infants born to mothers with acute HIV infection during pregnancy or breastfeeding have approximately double the risk of acquiring HIV compared with infants born to women previously diagnosed with HIV [3]. Transmission rates among this group of infants are as high as 36% [4]. The World Health Organization (WHO) recommends a four-pronged strategy to prevent HIV vertical transmission, which includes the prevention of HIV infection among women of reproductive age [5]. In Africa, detection of HIV infection in women in late pregnancy or in the postpartum period is uncommon as testing practices often focus on testing at first antenatal visit.

Data on socio-demographic and behavioural risk factors associated with acute HIV infection during pregnancy and postpartum period are limited. In Kenya, the region and employment of the participant were independent risk factors [6]. A study in Zimbabwe looking at socio-demographic and behavioural risk factors showed time of seroconversion, level of education of the participant and age as important factors [7].

Mozambique has little data on acute HIV infection and associated transmission risk factors, or on HIV incidence among pregnant and breastfeeding women. The country has a population of 22 million inhabitants, of which an estimated 25% are women of reproductive age. Southern Mozambique is highly affected by the HIV epidemic with a prevalence of 20% in women aged 15–49 years [8] and 21% in pregnant women [9]. Mozambique's Ministry of Health (MOH) established a national program for prevention of mother-to-child transmission (PMTCT) in 2002. By the end of 2010, 86% of health facilities with antenatal care (ANC) provided PMTCT services free-of-charge [10]. At the time of the study, the program focused on “opt-out” HIV testing offered to all pregnant women at the first ANC visit, repeat HIV testing at delivery, and testing women of unknown status at any Mother-and-Child Health (MCH) entry point postpartum. Partner testing is available and encouraged, though uptake remains low.

The 2011 Mozambique national vertical transmission rate among infants less than or equal to two months was 7.4% with an 18-month transmission rate of 19% [10]. The additional infant infections reflect both on-going transmissions through breastfeeding in known HIV-positive women as well as in women with incident HIV infection during this period.

With the emphasis on preventing transmission to infants born to HIV-positive women, primary prevention for women identified as HIV-negative early in pregnancy is often neglected. This is a missed opportunity to reduce the incidence of HIV in women aged 15–49 by 50% and to meet the target of the Global Plan for the elimination of paediatric HIV [11].

The Elizabeth Glaser Pediatric AIDS Foundation (EGPAF) has supported the national PMTCT program in Mozambique since 2004. A prospective study was conducted to estimate HIV incidence and associated risk factors among HIV-negative postpartum women in Southern Mozambique.

## Methods

### Study design

A prospective cohort study was conducted between March 2008 and July 2011 in six rural primary health care facilities in Gaza and Maputo provinces. Health facilities were selected by convenience sampling, based on the high HIV prevalence and a high attendance in MCH units. Study staff recruited HIV-negative women attending MCH services in maternity wards, postpartum care and family planning clinics. Enrolled women were followed quarterly until 18 months postpartum.

### Study population and procedures

Women aged ≥18 years who delivered in a study health facility or attended a postnatal visit within two months of delivery, and tested HIV-negative at that time, were invited to participate. HIV screening was performed per national guidelines with the Determine HIV-1/2^®^ (Abbott Laboratories, Wiesbaden, Germany) assay. The informed consent process was conducted in Portuguese or Changana (the local language), depending on the participants’ preference. Only participants who provided written consent were enrolled in the study.

At enrolment, participants responded to a questionnaire administered by a trained study counsellor. The questionnaire included socio-demographic information and questions regarding their sexual activity, condom use, occurrences of STIs, information about their husband/partner, and knowledge of HIV and PMTCT. A clinical examination was performed by an MCH nurse.

Study follow-up visits were scheduled at 3, 6, 9, 12, 15 and 18 months postpartum. At each visit, the study counsellor administered a questionnaire, women were counselled on HIV prevention and testing, and a nurse performed a clinical examination and HIV rapid antibody testing. Whole dried blood spot (DBS) samples were collected and stored for later confirmatory testing for all women with newly identified HIV infection.

HIV-negative women were counselled and reminded of their next study visit. Women who recently tested positive to HIV received counselling on their result, were referred for further HIV care and treatment and follow-up of their HIV-exposed infant, and were then discharged from the study. Results of the initial HIV DNA-PCR testing of infants of newly diagnosed women, when available, were extracted from routine PMTCT program records.

HIV DNA-PCR analysis (Roche Amplicor HIV-1 DNA Test, version 1.5, Roche Molecular Diagnostics, Branchburg NJ, USA) was performed on all DBS samples from women who seroconverted to confirm HIV infection and to estimate the time of infection. At HIV diagnosis, blood was taken for CD4-cell count (FACSCount or FACSCalibur, both from Becton Dickinson, San Jose, CA, USA) and HIV RNA viral load testing (Cobas AmpliPrep/Cobas TaqMan HIV-1 test, version 2.0, Roche Molecular Systems, Inc.). All DBS storage, and HIV DNA and RNA testing was done at *Instituto Nacional de Saúde*, Maputo.

Several strategies were implemented to retain women: 1) participants were reimbursed for transport; 2) study counsellors collected contact details with permission of the participant and traced those who did not show up for one month after the scheduled visit; and 3) the study ID was written on the child health card for participant identification at vaccination clinics and referral to the study counsellor. Reasons for lost to follow-up (LFU) were explored using the tracing registers.

### Ethics

The protocol was approved by Mozambique's National Health Bioethics Committee. Written informed consent was obtained from all participants.

### Data collection and statistical analysis

Collected variables included clinical (e.g. sexually transmitted infection), laboratory (e.g. HIV test results), socio-demographic (e.g. age, marital status, educational level), knowledge of HIV/PMTCT and sexual risk behaviour. Double data entry was done using EpiData Version 1.1. Statistical analyses were generated using SAS/STAT software, Version 9.2 of the SAS System for Windows (SAS Institute Inc., Cary, NC, USA).

Seroincidence rates were calculated using the number of new HIV infections per 100 women-years at risk, based on women's exposure time. For non-seroconverters, the exposure time was defined as the interval between enrolment and the last HIV test. For seroconverters, the exposure time was calculated as the interval between enrolment and estimated time of HIV infection, defined as the midpoint between the last negative test result and the first positive HIV test result.

Seroincidence rates were compared with women's baseline socio-demographic characteristics, knowledge on HIV/PMTCT and sexual behaviours employing bivariable Poisson regression models using one exposure with one response. The HIV vertical transmission rate among newly diagnosed women was calculated as the proportion of HIV-infected infants over the total number of infants with an initial HIV test after detection of maternal seroconversion. Using the regional annual birth rates, vertical transmission rates for women within and out of the PMTCT program and our results on incidence and vertical transmission rate, the contribution of postpartum HIV incidence to vertical transmission was estimated.

## Results

**Figure 1:**
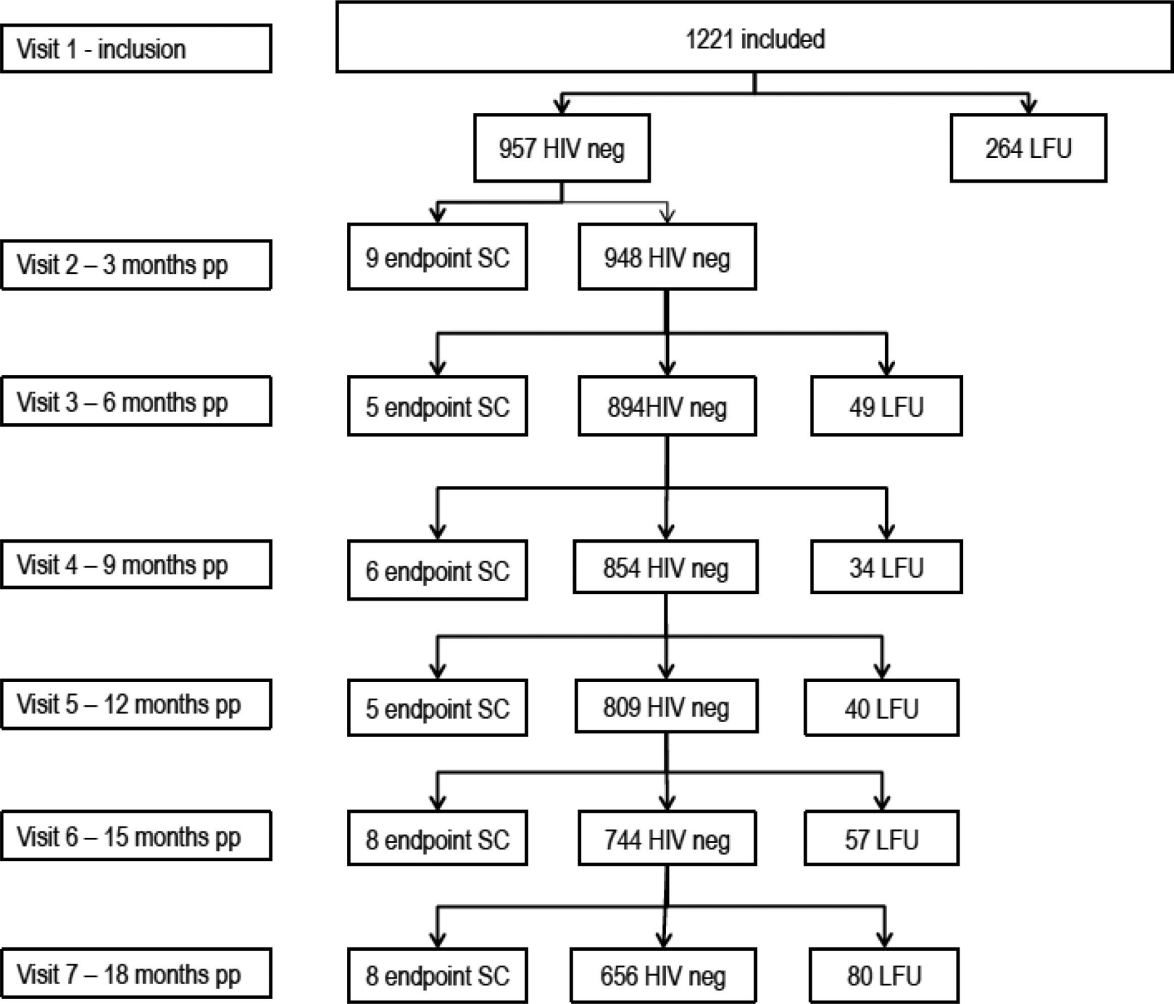
Flow of participants during the study. SC, seroconversion; LFU, lost to follow-up; pp; postpartum; neg, negative.

A total of 1221 women were enrolled in the study. Eleven women were enrolled outside of the inclusion period, with a median time of enrolment of 63 days (Interquartile Range (IQR) 61–89) postpartum, compared to a median of one day (IQR 1–4) of enrolment of the others. After enrolment, 957 (78%) women had at least one follow-up visit and were included in the analysis (Figure 1). The median follow-up period for women in the analyzed cohort was 18.2 months (IQR 15.3–19.2) with a total follow-up time of 1278 women-years.

Despite the tracing mechanism that was put in place, only 664 (54%) women had a last follow-up visit at 18 months. The average LFU rate per visit in the analyzed cohort was 8.8%. Main reasons for loss to follow-up included: incorrect address, traveling, change in address and refusal by participant or family member.

### Baseline characteristics

Table 1 shows the basic characteristics of the whole cohort with disaggregated comparison of women with and without any follow-up visits after enrolment visit.

**Table 1:** Baseline characteristics of all participants included in the study (*n*=1221), by the presence of any follow-up visit

	Total	No follow-up visit (N)	Follow-up visit (N)	Chi-square test (*p*-value)
Number (total)	1221	264	957	–
Province	–	–	–	**<0.0001**
Maputo	600	101 (38%)	499 (52%)	–
Gaza	621	163 (62%)	458 (48%)	–
Age (years)	–	–	–	–
Mean (±sd)	25 (19–31)	25 (18–32)	25 (19–31)	–
Median (IQR)	24 (20–29)	24 (20–29)	24 (20–29)	–
Missing	23	13	10	–
Age category (years)	–	–	–	0.2
18–19	213	49 (19%)	164 (17%)	–
20–24	420	79 (30%)	341 (36%)	–
25–29	280	62 (23%)	218 (23%)	–
30–34	167	29 (11%)	138 (14%)	–
> 34	118	32 (12%)	86 (9%)	–
Missing	23	13 (5%)	10 (1%)	–
Parity	–	–	–	0.4
< 3	566	111 (42%)	455 (48%)	–
≥ 3	628	135 (51%)	493 (51%)	–
Missing	27	18 (7%)	9 (1%)	–
Age at first child	–	–	–	–
Mean (±sd)	18 (16–20)	18 (13–23)	19 (17–21)	–
Median (IQR)	19 (17–20)	18 (17–19)	18 (17–20)	–
Missing	227	64	163	–
Marital status	–	–	–	0.2
Married/living with partner	851	186 (70%)	665 (70%)	–
Divorced/separated/widow/single	241	41 (16%)	200 (21%)	–
In relationship but not living with partner	118	28 (11%)	90 (9%)	–
Missing	11	9 (3%)	2 (0%)	–
Polygamy	–	–	–	0.2
No polygamous marriage	657	147 (56%)	510 (53%)	–
Polygamous marriage	119	33 (12%)	86 (9%)	–
Missing	445	84 (32%)	361 (38%)	–
Educational level	–	–	–	0.2
Less than primary education	799	182 (69%)	617 (64%)	–
Primary education or higher	422	82 (31%)	340 (36%)	–
Partner educational level	–	–	–	**0.01**
Less than primary education	536	129 (49%)	407 (43%)	–
Primary education or higher	495	88 (33%)	407 (43%)	–
Missing	190	47 (18%)	143 (15%)	–
History of professional life	–	–	–	0.5
Never had a job	1016	210 (80%)	806 (84%)	–
Ever had a job	184	42 (16%)	142 (15%)	–
Missing	21	12 (4%)	9 (1%)	–
Partner employment	–	–	–	**<0.0001**
Unemployed	211	44 (17%)	167 (717)	–
Own business	184	35 (13%)	149 (16%)	–
Employee	247	40 (15%)	207 (22%)	–
Mineworker	330	106 (40%)	224 (24%)	–
Others	116	15 (6%)	101 (10%)	–
Missing	133	24 (9%)	109 (11%)	–

Boldfaces means significant (p<0.05).

The median age of participants at enrolment was 24 years (IQR 20–29). Seventy percent of the women (851/1221) were married or lived permanently with their partner. Average education level among women was low: 66% (799/1221) had no education or did not finish primary education. The median age of first childbearing was 19 years (IQR 17–20). At 12- and 18-month follow-up visits, 16/639 (2.5%) and 246/661 (37%) respondents, respectively, said they had stopped breastfeeding.

Women with no follow-up visits after enrolment differed significantly from women in the analyzed cohort in province, partners’ level of education and type of employment. Participants retained in the cohort to the end of follow-up differed in the same variables (*p*<0.0001, *p*=0.01 and *p*<0.0001 respectively) from those lost during the follow-up period (data not shown).

### HIV incidence

During the follow-up period, 41 incident HIV infections were identified among the 957 women with at least one follow-up visit. HIV diagnosis was confirmed by DNA-PCR in all but one case due to lack of a biological specimen.

The overall incidence among women during postpartum period was 3.20 per 100 woman-years (95% CI: 2.30–4.46). The highest incidence of 4.92 (95% CI: 2.65–9.15) was seen in the youngest age group of 18–19 years, with decreasing incidence with older age. Incidence was 3.39 (95% CI: 2.08–5.53) between 20 and 24 years; 3.49 (95% CI: 1.88–6.49) between 25 and 29 years; 2.16 (95% CI: 0.81–5.76) between 30 and 34 years and 0.84 (95% CI: 0.12–5.93) above 34 years. The median age at study enrolment of women who seroconverted was 22 (IQR 20–25) years.

Of all seroconversions, 14 (34%) were identified during the first six months postpartum, 11 (27%) between 6 and 12 months and 16 (39%) occurred in the period 12–18 months postpartum. The median time from enrolment to time of serological diagnosis of HIV was 299 days (IQR 183–489).

The incidence was slightly but not significantly higher in Gaza Province (3.62; 95% CI: 2.36–5.55) than in Maputo Province (2.86; 95% CI: 1.84–4.43).

Participants with HIV seroconversion were referred for further clinical care and staging, CD4-cell counting and antiretroviral treatment (ART), if eligible. The median CD4-cell count among 24 seroconverters tested was 604 cells/µl (IQR 429–820) and the median viral load was 22,378 copies/ml (IQR 8,131–113,497) (*n*=26). Six participants refused blood collection for CD4-cell count at the time of seroconversion and one result was lost. Results of 10 participants were not available to the study staff due to referral of participants from a study site to another health facility.

### Risk factors for seroconversion

Incidence was significantly higher in women whose partners had a higher educational level (*p*=0.01) and among women with fewer children (*p*=0.03) (Table 2). Being in a relationship but not living with the biological father doubled the risk for HIV infection, compared to being married or living together. Younger women were more at risk: for every one-year increase in age, incidence decreased 0.93 per woman-year (95% CI: 0.87–0.99; Table 2).

**Table 2:** Comparison of HIV incidence rates for demographic risk factors using bivariable Poisson regression models (*n*=957)

	Number	Number SC	Risk ratio (95% CI)	*p*-value*
Province	–	–	–	–
Maputo	499	20	–	–
Gaza	458	21	1.27 (0.69–2.34)	0.45
Age (years)	947	41	0.93 (0.87–0.99)	**0.02**
Age category (years)	–	–	–	–
18–19	164	10	–	–
20–24	341	16	0.67 (0.31–1.52)	0.36
25–29	218	10	0.71 (0.30–1.71)	0.44
30–34	138	4	0.44 (0.14–1.40)	0.16
> 34	86	1	0.17 (0.02–1.33)	0.09
Marital status	–	–	–	–
Married or living together	665	24	–	–
Divorced/separated/widow/single	200	10	1.37 (0.66–2.87)	0.40
Relationship but not living with partner	90	7	2.13 (0.92–4.95)	0.08
Polygamous marriage	–	–	–	–
No	508	17	–	–
Yes	86	5	1.75 (0.64–4.73)	0.27
Number of children	–	–	–	–
< 3	455	26	–	–
≥ 3	493	14	0.48 (0.25–0.93)	**0.03**
Educational level	–	–	–	–
Less than primary education	617	26	–	–
Primary education or higher	340	15	0.99 (0.52–1.86)	0.96
Partner educational level	–	–	–	–
Less than primary education	407	10	–	–
Primary education or higher	407	22	2.10 (1.01–4.50)	**0.05**
Ever had a job	–	–	–	–
No	806	34	–	–
Yes	142	7	1.14 (0.50–2.57)	0.76
Partner employment	–	–	–	–
No job	167	6	–	–
Employee	208	3	1.71 (0.61–4.80)	0.31
Mineworker	225	8	0.38 (0.09–1.51)	0.17
Other	101	5	1.02 (0.35–2.93)	0.98
Own business	149	9	1.23 (0.36–4.03)	0.73
Partner works out >4 weeks	–	–	–	–
No	604	28	–	–
Yes	324	10	0.67 (0.32–1.38)	0.27

SC, seroconversion; CI, confidence interval.

* *p*-values from Poisson regression models (i.e. generalized linear models using the Poisson distribution with the log link and an offset accounting for person-years).

Boldfaces means significant (p<0.05).

Ninety eight percent of the respondents had heard of HIV and AIDS (Table 3) and 99% of the participants had heard about vertical HIV transmission. When asked how women transmit infection to babies, 28% of women mentioned all three modes of vertical transmission. Those who did not identify transmission via breast milk had about a two-fold increased risk of infection (*p*=0.06; Table 3).

**Table 3:** Comparison of HIV incidence rates for baseline knowledge-based risk factors using bivariable Poisson regression models (*n*=957)

	Number	Number SC	Risk ratio (95% CI)	*p*-value*
Ever heard of AIDS?	–	–	–	–
No	22	2	–	–
Yes	929	38	0.43 (0.10–1.77)	0.24
Ever heard of vertical transmission?	–	–	–	–
No	10	1	–	–
Yes	930	40	0.47 (0.06–3.40)	0.45
Identification of risk of vertical transmission through pregnancy	–	–	–	–
No	387	20	–	–
Yes	479	18	0.70 (0.37–1.32)	0.27
Identification of risk of vertical transmission through delivery	–	–	–	–
No	462	20	–	–
Yes	404	18	1.03 (0.54–1.94)	0.93
Identification of risk of vertical transmission through breastfeeding	–	–	–	–
No	100	8	–	
Yes	766	30	0.47 (0.22–1.03)	0.06
Is there treatment for HIV?	–	–	–	–
No	7	0	0 (0–∞)	>0.999
Yes	896	37	–	–
Is there cure for HIV?	–	–	–	–
No	666	28	–	–
Yes	197	6	1.43 (0.64–3.32)	0.39
Can transmission be decreased by condom use?	–	–	–	–
No	16	0	0 (0–∞)	>0.999
Yes	903	37	–	–

SC, seroconversion; CI, confidence interval.

* *p*-values from Poisson regression models (i.e. generalized linear models using the Poisson distribution with the log link and an offset accounting for person-years).

Among sexual/behavioural risk factors collected at the time of enrolment, only having sex with a partner outside of their relationship increased HIV risk significantly (*p*=0.001, Table 4). Women with sexual debut before 17 years of age were at almost twice-higher risk (*p*=0.13) but this was not statistically significant.

**Table 4:** Comparison of HIV incidence rates for sexual/ behavioural-type risk factors using bivariable Poisson regression models (*n*=957)

	Number	Number SC	Risk ratio (95% CI)	*p*-value*
Age at first sexual activity (years)	713	31	0.84 (0.69–1.03)	0.09
Age at first sexual activity (years)	–	–	–	–
≤ 16	377	20	–	–
> 16	336	11	0.57 (0.27–1.19)	0.13
Self risk assessment	–	–	–	–
No risk	42	1	–	–
Medium risk	351	15	1.88 (0.24–14.23)	0.54
High risk	59	9	1.91 (0.24–15.12)	0.54
Ever tested for HIV?	–	–	–	–
No	93	4	–	–
Yes	837	34	0.94 (0.33–2.65)	0.91
Knowledge if partner was ever tested for HIV	–	–	–	–
Was not tested	546	21	–	–
Was tested	232	8	0.85 (0.38–1.92)	0.70
Don't know if was tested	151	10	1.74 (0.82–3.70)	0.15
Condom use with partner	–	–	–	–
Never	757	28	–	–
Sometimes	172	11	1.73 (0.86–3.47)	0.12
Always	5	0	0 (0–∞)	>0.999
Ever had sex with other person than partner?	–	–	–	–
No	922	39	–	–
Yes	4	2	16.91 (3.91–67.05)	**<0.001**
Knowledge if partner had ever sex with others	–	–	–	–
No	297	13	–	–
Yes	133	7	1.13 (0.45–2.84)	0.79
Ever used something to dry vagina?	–	–	–	–
No	685	29	–	–
Yes	232	11	1.08 (0.54–2.15)	0.84
Ever experienced domestic violence by partner?	–	–	–	–
No	863	38	–	–
Yes	87	3	0.80 (0.25–2.59)	0.71
Ever experienced sexual violence by partner?	–	–	–	–
No	919	40	–	–
Yes	29	1	0.68 (0.09–4.98)	0.71
Treated for STD at first visit	–	–	–	–
No	759	34	–	–
Yes	12	0	0 (0–∞)	0.9997

SC, seroconversion; CI, confidence interval.

*p-values from Poisson regression models (i.e. generalized linear models using the Poisson distribution with the log link and an offset accounting for person-years).

The values in bold are the ones that are significant (p<0.05).

### Infant outcomes

Data on the initial HIV testing of infants of women who seroconverted were available in routine records for 29 infants. One child died before maternal seroconversion was detected; the remaining 11 mother–infant pairs were LFU before an infant DBS sample was collected. The median time from identification of maternal seroconversion to infant DBS sampling was 52 days (IQR 0–99). Thirteen infants were tested the same day as the maternal HIV diagnosis. The median age of infants at the time of HIV testing was 12.6 months (IQR 8.9–17.0). Six of the 29 tested infants were HIV-positive (21%; 95% CI: 5–36). Five HIV positive infants were tested on the same day as the maternal HIV diagnosis and one was tested two months after maternal diagnosis. All HIV-infected infants were referred for ART initiation according to the national guidelines. No further infant follow-up information was available.

Maternal CD4-cell count was associated with infant infection at first infant HIV test: among 16 women with a CD4-cell count>350 cells/µl and known infant infection status, two had HIV-infected infants, while both infants from the two mothers with a CD4-cell count ≤350 cells/µl were HIV-infected (Fisher's Exact test, *p*=0.006). No significant association was seen between maternal viral load and infant HIV status: none of the six infants of mothers with a viral load ≤10,000 copies/ml and six infants of 23 mothers with a viral load>10,000 copies/ml were HIV infected (Fisher's Exact test, *p*=0.168).

### Estimation of the effect of postpartum HIV incidence on HIV infections in children

With the postpartum HIV incidence of 3.20 per 100 women-years and a 21% vertical transmission rate in this study, we estimated the proportion of HIV-infected children attributable to postpartum seroconversion. With an annual estimated number of births of 93,718 in Gaza and Maputo provinces [12] and a HIV prevalence in pregnancy of 21% [9], 19,681 mothers will be HIV-positive at time of delivery. For those who have access to health facilities (58% of pregnant women [13] and the estimated postpartum vertical transmission rate within the PMTCT program of 12% [10], 1,370 infants are estimated to be HIV-infected. Women without access to services have an increased risk of transmission of 31% [14], resulting in 2,562 HIV-infected infants. With 3.2 incident infections per 100 women-years, 3,554 of the 74,037 HIV-negative mothers at delivery will be infected during the first 18 months postpartum. With a postpartum vertical transmission rate of 21%, 746 infants are estimated to become infected – thus, 16% (746/4,678) of the HIV-infected infants are due to a postpartum incident infection in women (see Supplementaryfile).

## Discussion

Our study shows a high HIV incidence (3.20 per 100 women-years) in women during the postpartum period in a high prevalence region of Mozambique, with the subsequent transmission to infants accounting for one in six HIV-infected infants. This first description of HIV incidence in this population is consistent with studies in similar populations in other sub-Saharan African countries [14–19]. Our study confirms an active epidemic in the Southern region of the country, which was suggested in the epidemic trend analysis conducted in 2009 [9] using the Estimation and Projection Package [20].

The high incidence among postpartum women aged 18–19 years (4.92 per 100 women-years), is consistent with results from Zimbabwe [21] and non-pregnant young women (≤24 years) in Kwazulu-Natal [22]. However, in a recent study among 18–24 year olds with higher education levels in Maputo City, an overall incidence of 1.14 per 100 person-years (95% CI: 0.88–2.51), and 1.49 per 100 person-years (95% CI: 0.67–1.92) in female youth was observed (Viegas, personal communication, 2013). Factors associated with this considerable difference, such as pregnancy, rural or urban setting, education, or other social factors should be further explored. As young people are at a high risk of HIV infection, sexual education and integrated HIV education and prevention programs in the primary and secondary schools are important in the efforts to reduce risk behaviour.

Although a small number of infants were tested, a vertical transmission rate among seroconverters of 21% was seen, similar to findings from other studies with estimated rates higher than 20% [3, 14]. However, this needs to be interpreted with caution given the small numbers, wide CI and missing infant HIV status results for 12 infants. The vertical transmission rate would range from 15% to 44% if none or all of these 12 infants were considered HIV-infected, respectively. The long breast feeding period and considerable number of women who were still breastfeeding at 18 months indicates continued risk of additional infant transmission.

Early identification of incident infection in postpartum women and immediate testing of their children is critical for initiating ARV prophylaxis to prevent infection in infants found to be uninfected and initiating ART for HIV-infected infants. While HIV disease progression in postnatally infected infants has been associated with slower disease progression than perinatally infected infants [23], current WHO guidelines recommend ART for all children under five [24].

At the time of the study, Mozambique followed the 2006 WHO PMTCT guidelines that did not include postpartum antiretroviral prophylaxis. With the recent implementation of PMTCT Option B+, offering lifelong ART to all HIV-positive pregnant and lactating women [25], it is expected that the overall risk of vertical transmission will decrease significantly. Routine regular repeat HIV testing for negative postpartum women will be required to realize the benefits of Option B+ for women with acute HIV infection, especially since 66% of the seroconversions occurred after six months postpartum. Given the time that it takes for ART to decrease viral load, immediate nevirapine prophylaxis in infants may decrease the transmission risk further.

Repeat testing strategies will not be sufficient to reduce the number of infant infections due to maternal postnatal incidence as infant infection is likely to occur before the diagnosis of infection in the mother and retention of HIV-negative women in postpartum care is a challenge. Therefore focused attention on comprehensive primary prevention for all HIV-negative women in antenatal and MCH settings is critical, and often lacking. This includes HIV testing of partners of HIV-negative women, which is critical for identification of discordant couples and intervention with ART for the partner or pre-exposure prophylaxis for the woman to decrease the chance of transmission [24, 26]. Associations between partner characteristics and HIV incidence in postpartum women emphasize the need to involve both partners in primary prevention efforts.

There is clear benefit of early ART on prevention of HIV infection [27, 28]. Expansion of ART programs throughout Mozambique and other African countries should also contribute to a reduced incidence of HIV in postpartum women and subsequently in their infants. Mozambique's HIV policy provides universal access to ART for HIV-positive partners of HIV-negative pregnant women, which should contribute to a decrease in incidence.

However, partner testing remains low. The Southern provinces of the country are known for their migrant population, with a great number of men working abroad. In our study, 27% of women had partners who were mineworkers in South Africa. Moreover, the Southern region has a cultural paternal tradition that hinders male involvement in reproductive health.

Counselling and testing points at bus stops of migrant workers, working with the companies to provide employee testing, testing campaigns during the holiday season in the community or at gathering places are strategies to be evaluated to increase both partner testing and periodic re-testing of postpartum women.

Our research had several limitations: the differential high loss to follow-up over time could lead to biased incidence estimates, such as an overestimation due to higher retention of women with partners with a higher education, which is associated with an increased risk of incident infections. Multivariate Poisson regression analyses were not possible to perform with sufficient power due to the low number of incident HIV infections. Similarly, there was insufficient power to conduct a longitudinal analysis comparing knowledge, sexual/behavioural risk factors over time due to low or irregular follow-up. With the small number of infants tested, transmission rates need to be interpreted with caution. There is also a risk of reporting bias, as women report on their and their partners’ behaviour. Finally, we cannot extrapolate the results to other regions in Mozambique due to different epidemic profiles and socio-culturally different populations.

## Conclusions

The high incidence of HIV in postpartum women in Southern Mozambique and similar African populations underscores the importance of effective primary prevention strategies targeting breastfeeding women and their partners in the elimination of paediatric HIV efforts. Achieving the “Global Plan” targets [11] will require a comprehensive combination prevention strategy with community, behavioural and biomedical interventions, such as pre-exposure prophylaxis during breastfeeding, adapted to these vulnerable populations in high prevalence settings. Tracking HIV incidence over time will be critical for monitoring the effects of current prevention and expanding ART programs.
